# Barriers and Facilitators to 24-Month Maintenance of the Faith, Activity, and Nutrition Program in the U.S.

**DOI:** 10.1007/s10943-024-02012-9

**Published:** 2024-03-25

**Authors:** Kelsey R. Day, John A. Bernhart, Sara Wilcox

**Affiliations:** 1 Prevention Research Center, Arnold School of Public Health, University of South Carolina, Columbia, SC USA; 2 Department of Exercise Science, Arnold School of Public Health, University of South Carolina, Columbia, SC USA; 3 Department of Health Promotion, Education, and Behavior, Arnold School of Public Health, University of South Carolina, Columbia, SC USA

**Keywords:** Faith-based, Churches, Physical activity, Nutrition, Church leaders

## Abstract

Little is known about the barriers and facilitators to organizational maintenance of faith-based health promotion programs. This study used qualitative data (collected from 2016–2019) from pastors (*n* = 81) and program coordinators (*n* = 103) to identify barriers and facilitators to 24-month maintenance of a faith-based physical activity (PA) and healthy eating (HE) intervention in South Carolina. Barriers differed for PA versus HE: resistance to change impeded HE while church characteristics tended to impede PA. Similar themes emerged for PA and HE facilitators: healthy opportunities, church communication, and consistency. Future research should build upon this study to tailor faith-based health promotion programs for long-term sustainability.

## Introduction

Faith-based organizations are promising partners for health promotion interventions as groups at-risk of chronic disease, namely older adults, racial minoritized individuals, and rural residents, report regular church attendance (Pew Research Center, 2019). Existing research has demonstrated the success of faith-based health promotion interventions in improving individual-level health behaviors such as physical activity (PA) and healthy eating (HE) (Bopp et al., 2012; Dunn et al., 2021; Parra et al., 2018). The success of these interventions may be attributed to the role churches play in their communities as trusted sources of information and as gathering places for their members (Campbell et al., 2007). A growing number of churches seek to connect physical and spiritual wellbeing through regular heath-focused messaging or events, and many have health ministry committees (Austin & Harris, 2011). Further, churches are broadly located across the U.S., including in rural and under resourced communities, which may expand the reach of faith-based health promotion programs. Therefore, churches are promising settings to promote overall health and to prevent chronic diseases.

Previous studies have found that the support of church leaders, particularly those in the Black Church, is influential to the success of health promotion programs (Baruth et al., 2015; Wilcox et al., 2013a, 2021). Qualitative research with African American pastors has revealed that they believe they can positively impact church member health, and that they view themselves as role models for health behaviors (Ammerman et al., 2003; Baruth et al., 2015). Despite the positive influence of church leaders, faith-based PA and HE programs experience common barriers to program implementation (Bernhart et al., 2019). Few studies to date have examined such barriers and facilitators to long-term PA and HE program maintenance in faith-based settings.

The Faith, Activity, and Nutrition program (FAN) promotes PA and HE among church members by targeting policy, systems, and environmental change in churches (Wilcox et al., 2010, 2013a, 2013b, 2018). The FAN training assists churches in increasing PA and HE opportunities, increasing PA and HE messaging, creating PA and HE policies, and increasing pastor support for PA and HE. As a part of FAN, a person within the church serves as the program coordinator and liaison with the study team (i.e., FAN Coordinator). The support of church pastors is also an instrumental part of the program’s success. The evaluation of the FAN program is guided by the Reach, Effectiveness, Adoption, Implementation, Maintenance (RE-AIM) framework (Glasgow et al., 1999). Maintenance in the long-term is a critical indicator of an organization’s institutionalization of an intervention and speaks to the likelihood of lasting positive change. A 2015 systematic review of 82 studies that used at least one dimension of the RE-AIM framework concluded that long-term (24 month) evaluation of maintenance is rare (Harden et al., 2015). A separate 2021 review of faith-based PA and HE interventions using the RE-AIM framework similarly concluded that maintenance was the least frequently reported RE-AIM construct, and that maintenance at the organizational level was only reported by 8% of the included studies (Dunn et al., 2021).

Among the studies of faith-based PA and HE interventions that have reported organizational maintenance, there is a lack of conclusiveness on what factors impede or support church maintenance of intervention activities. For example, church coordinators in one study cited staffing concerns and low congregant interest as barriers to intervention maintenance at 6 months (Allicock, 2013); while, coordinators in a separate study reported their intent to maintain at 12 months because their congregants’ attitudes toward healthful food offerings had improved (Allicock et al., 2012). Of the two available studies that have examined organizational maintenance of faith-based PA and HE interventions beyond 12 months, both focused on the maintenance of intervention components and did not offer descriptive detail on barriers and facilitators to intervention maintenance (Wilcox et al., 2020, 2022). Therefore, the purpose of this paper is twofold: (1) to identify salient barriers and facilitators to 24-month organizational maintenance of FAN among Pastors and FAN Coordinators and (2) to describe church leaders’ support for maintaining FAN activities 12–24 months after training and church leaders’ intent to maintain FAN going forward.

## Methods

### Design and Recruitment

Data were drawn from the first and second phases of the FAN Dissemination and Implementation study. The first phase (Phase 1) was a group randomized controlled trial conducted in a rural and medically underserved county in South Carolina (SC). Complete descriptions of the Phase 1 study design, recruitment process, and implementation outcomes are available elsewhere (Saunders et al., 2019; Wilcox et al., 2018). Briefly, all churches in the county were invited to participate (*n* = 132) and were eligible if they had at least 20 members and were willing to accept their randomization status. Fifty-nine churches were randomized to early (*n* = 39) or delayed (*n* = 20) intervention; after withdrawals, 54 churches (36 early, 18 delayed) attended training. Training for early churches took place in 2015 while training for delayed churches took place in 2016. The one-day training was delivered by community health advisors (CHAs); three-five members from each church attended training, including a leader who was the designated FAN coordinator for the church. CHAs provided technical assistance to FAN coordinators (up to eight calls) and to pastors (up to four calls) during the 12-month implementation period (Sharpe et al., 2018). Quantitative data regarding 24-month maintenance of program implementation (Wilcox et al., 2020) as well as barriers to and facilitators of 12-month program implementation have been previously reported previously (Bernhart et al., 2019).

The second phase (Phase 2) was a quasi-experimental study in SC. Complete descriptions of the study design, recruitment process, and implementation outcomes are available elsewhere (Hutto et al., 2021; Wilcox et al., 2021). In brief, all churches in the SC Conference of the United Methodist Church (*N* = 985) were invited to participate in FAN. Churches were eligible if they had at least 20 regular attendees and if the pastor and a church leader (the FAN coordinator) agreed to complete evaluations at baseline, 12 months, and 24 months. Overall, 115 churches enrolled in the study and received training. There were instances in which a pastor served more than one church; therefore, one of his/her churches was randomly selected to be included in evaluation. Therefore, 93 distinct churches were included in evaluation activities. Leaders from eligible churches were trained by community health advisors (CHAs) during 2017–2018. CHAs also provided ongoing technical assistance to participating churches (Sharpe et al., 2020).

### Sample and Data Collection

The University Institutional Review Board reviewed the study protocols and deemed the study to have exempt status. Data presented in this paper are from the 24-month surveys completed by FAN coordinators and pastors conducted with the early intervention churches in Phase 1 and all churches in Phase 2. In Phase 1, 33/36 FAN coordinators and 28/36 pastors from trained early intervention churches completed the 24-month survey (34 churches represented by pastor or FAN coordinator). In Phase 2, 70/93 FAN coordinators and 53/93 pastors completed the 24-month survey (81 churches represented by the pastor or FAN coordinator).

Most surveys were administered via telephone, and responses to open-ended questions were typed into a computer-aided telephone interviewing (CATI) system by trained interviewers in a survey research center distinct from the research team. For non-responders, the survey research center left up to three messages (more attempts were made without leaving messages) across different days of the week and times of day. They left their toll-free number with extended evening hours to call back to complete the survey. After the second message, the survey research center shared the names of non-responders with the research team, who then reached out to the non-respondent via email and telephone before the survey research center left their final message. All non-responders were also sent a letter emphasizing the importance of completing the survey. Finally, the research team contacted FAN coordinators who completed the survey in instances where the pastor did not complete the survey to ask for their help in reaching out to the pastor to encourage completion (and vice versa). Online or paper surveys were offered to those who were not responsive or when requested with similar follow-up protocols for non-responders as described previously. The responses to open-ended questions typed or written by these individuals were used for analyses. All responses were exported from the CATI software into Excel.

At 24 months, FAN coordinators and pastors from both phases answered four open-ended questions regarding barriers to and facilitators of maintaining the PA (two questions) and HE (two questions) portions of the FAN program: “What are some things that helped your church to keep FAN-related activities that promote [physical activity OR healthy eating] going over the past year?” and “What are some things that made it hard for your church to keep FAN-related activities that promote [physical activity OR healthy eating] going over the past year?” (Bernhart et al., 2019) FAN coordinators and pastors also answered two questions regarding the support in their church for maintaining the PA (one question) and HE (one question) components of FAN between 12 and 24 months: “Over the past year, to what extent has your church supported FAN-related [healthy eating activities OR physical activities] or changes that promote [healthy eating OR physical activity]?” Church leaders answered using a 4-point scale, “The church has been resistant” (1), “There has been little or no support” (2), “There has been some support” (3), and “There has been strong support” (4). They also answered two questions regarding their likelihood of continuing PA (one question) and HE (one question) components in months 24–36: “How likely is it that your church will continue FAN or FAN-related activities to promote [healthy eating OR physical activity] in your church this coming year?” These questions were also answered using a 4-point scale, “Very unlikely” (1) to “Very likely” (4). For all questions, higher scores indicated greater support for FAN activities or likelihood of maintaining FAN activities. In both phases, FAN coordinators and pastors received a $20 gift card for completing 24-month surveys.

### Data Analysis

We compared pastor and FAN coordinator respondents with non-respondents, separately by phase, by the following church-level characteristics: church size (reported number of members as a continuous variable and categorized), predominant race of congregation, whether the pastor participated in training, and whether the church had a health ministry. Chi-square or Fisher’s exact tests were used for all categorical variables. A *t* test was used for reported number of members.

Responses from the four open-ended questions from the 24-month surveys from both phases were exported into Microsoft Excel for analysis. Two trained coders from the FAN research team (KD and JB) conducted a content analysis by independently categorizing and condensing statements into themes. Both coders used inductive category development techniques to categorize responses. After initial coding, the two coders discussed differences in theme categorizations and devised final codes. A third member of the research team (SW) then reviewed final codes to assure consistency. Discrepancies were discussed and resolved. Responses for each code were then ordered by the frequency with which each code appeared. We chose to focus on themes that emerged from at least 10% of respondents for each question, as this appeared to be a natural break in the code frequency. We also examined whether theme patterns differed by phase and for PA and HE. Because there was overlap in themes across FAN coordinator and pastor responses, data are reported as combined perspectives from both church leaders.

To analyze churches’ support for maintaining FAN activities from 12 to 24 months after training and intent to maintain FAN in months 24 and beyond, descriptive analyses (number of respondents, number of responses by category, and means for all questions) were performed in SAS, version 9.4 for all four Likert-scale questions. Descriptive analyses are reported separately by phase and for pastors/FAN coordinators. We also examined whether church characteristics (church size, predominant race of congregation, whether pastor had been trained, and presence of a health ministry) related to each of these variables using point-biserial (dichotomous variables) and Pearson correlation coefficients (continuous variables).

## Results

Pastor and FAN coordinator survey completion was unrelated to all church-level variables examined (church size, predominant race of congregation, pastor trained, and health ministry) for both phases (analyses not shown; *p* values > 0.05). Of the 34 Phase 1 early intervention churches where either the pastor or FAN coordinator completed the 24-month survey, the most common denominations represented were Baptist (*n* = 16), followed by non-denominational (*n* = 9), and African Methodist Episcopal/African Methodist Episcopal Zion (*n* = 4). Most churches had predominantly Black/African American members (97%), 88% had less than 100 members, and 79% had pastors that completed the FAN training. In Phase 1, 26–28 pastors and 30–32 FAN coordinators responded to each of the four open-ended questions (Table 1). Of the 81 Phase 2 churches where either the pastor or FAN coordinator completed the 24-month survey, all were United Methodist (by design), 43% were had predominantly Black/African American members (57% predominantly white), 67% had 101–499 members, and 63% had pastors that completed the FAN training. In Phase 2, 50–52 pastors and 66–68 FAN coordinators responded to each open-ended question.

**Table 1:** Number of respondents who answered open-ended questions for barriers and facilitators at 24 months

Questions	Number of respondents					
	Pastors (Phase 1)	FAN Coordinators (Phase 1)	Phase 1 Total	Pastors (Phase 2)	FAN Coordinators (Phase 2)	Phase 2 Total
1. What are some things that helped your church to keep FAN-related activities that promote healthy eating going over the past year?	27	30	57	50	66	116
2. What are some things that made it hard for your church to keep FAN-related activities that promote healthy eating going over the past year?	26	31	57	52	68	120
3. What are some things that helped your church to keep FAN-related activities that promote physical activity going over the past year?	27	32	59	50	67	117
4. What are some things that made it hard for your church to keep FAN-related activities that promote physical activity going over the past year?	28	32	60	51	67	118

### Barriers

In general, church leaders in both phases described different barriers to maintaining the PA versus the HE components of FAN (Table 2). Resistance to change emerged solely as a barrier to HE; while, church characteristics were more often cited as barriers to PA. Lack of participation/motivation, lack of time/conflicting priorities, and nothing (no barriers) were common barriers to both PA and HE maintenance. In Phase 2 only, themes related to leadership (lack of leadership, staff transition, and lack of internal support) were described as barriers to both PA and HE.

**Table 2:** Themes and select responses to physical activity and healthy eating maintenance barriers

Theme	Physical activity barriers (select quotations)	Ph 1 Count	Ph 2 Count	Healthy eating barriers (select quotations)	Ph 1 Count	Ph 2 Count
Resistance to change	*Not a prominent theme for physical activity*			Old people want to eat what they like, what's easier	17	28
				Low country bad eating habits, greasy foods		
Church characteristics	The congregation is older and does not show much interest in activities	16	34	The age of the congregation makes it hard to convince them to change their lifestyles	4	19
	Some are not physically able to participate because of the older congregation					
	Space and time requirements in church, utilizing indoor space, weather backups					
Lack of participation/motivation	Not many want to come to extra events for physical activities at church; most do their own thing. Our space and available days are limited	16	33	Participation of others. Not wanting to substitute or completely remove homemade cakes, pies, and sugary drink during special occasions like church anniversary	11	18
	Apathy, people do not care					
	They slowed down on every Sunday, and they got tired of the routine, the elderly were not wanting to move and do much. Makes it difficult to include them, had to work around them					
Lack of time/conflicting priorities	Sometimes it did not feel practical to incorporate activities at events such as meetings	11	27	People go home after church and are unable to attend activities with FAN	6	23
	Scheduling conflicts. Quite often events/meetings conflict with what needs to be done as far as physical activity is concerned			Trying to get the time, people's schedules are varied, hard to work in a group		
Nothing (no barriers)	As a whole they had support, nothing made it real hard	9	8	We didn't have any challenges, always healthy food options at each meal	14	14
				None, because really the ones that were on the committee, we were the ones who worked in the kitchen. So it's not hard for us to do. We just go by the FAN book—we read that book. We might cook with a little salt, but do not have salt shakers in the church. We have sodas sometimes, but it's diet sodas and water and lemonade. And we always serve some kind of fruit		
Lack of leadership (Phase 2 only)	A change in the senior pastor has made it difficult, when the church was already hesitant and not supportive anyway. The prior senior pastor was a major driving force, and the current vision moving forward for ministry does not place much, if any, emphasis on FAN-related ministry	4	17	Our FAN coordinators stopped after the previous pastor left; FAN was her effort, but did not appear to carry much Church support	3	14
	Someone to coordinate an evening exercise activity. There are a few people who have expressed an interest but no one has stepped forward to be the “leader.” We will be working on this during 2019–20			We had a complete change in leadership, from head pastor down. FAN disappeared as a result		

Themes were considered prominent if they featured in more than 10% of responses for each question. Ph 1 = Phase 1. Ph 2 = Phase 2

#### Resistance to Change

Resistance to change referred to individual or group unwillingness to accept new processes. The theme emerged as a prominent barrier to HE maintenance in both phases, but not to PA maintenance. When describing resistance to change, church leaders spoke about engrained eating habits and preferences among members that were difficult to overcome. Church leaders also described a cultural resistance to change in the church as a whole. For example, many leaders mentioned that the FAN HE guidelines differed from favored dietary traditions associated with the church’s geographic location. Multiple leaders cited the difficulty that members had abstaining from unhealthy foods traditionally associated with Southern cuisine, such as fried chicken (“Low country bad eating habits, greasy foods.”).

#### Church characteristics

Demographic characteristics of congregations and physical characteristics of churches were represented in leader responses as barriers to PA in two ways: age of members and available facilities. The most referenced congregation characteristic was the age of members, specifically older members. Leaders described a hesitance among older members to engage in PA due to health concerns, physical limitations, or lack of interest (“Some are not physically able to participate because of the older congregation.”). Leaders also cited a lack of facilities for physical activities at church as an impediment to hosting PA programs during inclement weather. When congregation characteristics appeared as a barrier to HE (Phase 2 only), it was often in reference to the age of members and the unwillingness to change eating habits among older individuals (“The age of the congregation makes it hard to convince them to change their lifestyles.”).

#### Lack of Participation/Motivation

The theme of lack of participation/motivation dealt with member/congregation unwillingness or disinterest to participate in the FAN program. It appeared most frequently in responses describing waning church member engagement in PA and HE-focused programming. For HE, leaders described a lack of motivation to eat healthier among members as well as difficulty maintaining a commitment to HE. For PA, leaders described how some members did not feel capable of participating due to physical limitations. For example, one church leader shared that older members did not want to take part in PA breaks or events due to tiredness (“They slowed down on every Sunday, and they got tired of the routine, the elderly were not wanting to move and do much. Makes it difficult to include them, had to work around them.”) Pastors and FAN coordinators also described an apathy among members for PA programming, as well as a general lack of participation in PA events held at church.

#### Lack of Time/Conflicting Priorities

In general, lack of time/conflicting priorities referred to church schedule constraints that made the continual incorporation of PA and HE challenging. Church leaders cited a lack of time to devote to PA and HE programming as well as individual time constraints that prohibited members from participating in extra programming. Pastors and FAN coordinators also described the difficulty of allotting extra time for physical activities in church, such as activity breaks during services or meetings (“Scheduling conflicts. Quite often events/meetings conflict with what needs to be done as far as physical activity is concerned.”) Other leaders mentioned that members preferred to be physically active at home and did not want to attend extra PA programs hosted at the church.

#### Nothing (No Barriers)

Many church leaders described a receptivity to FAN activities in their church and indicated that their church did not experience barriers to maintaining PA or HE. Church leaders stated they were able to integrate many FAN activities into existing programs or events and did not feel they faced barriers once activities became habitual. Multiple churches cited how they started adding healthy options to meals and making substitutions for unhealthy foods, which became a standard part of their food preparation process.

#### Lack of Leadership, Staff Transition, and Lack of Internal Support (Phase 2 Only)

Pastors and FAN coordinators in Phase 2 cited issues related to lack of leadership as barriers to both PA and HE maintenance. The lack of leadership and staff transition themes emerged as several leaders described staff (i.e., FAN Coordinator) or pastor turnover. Leaders most frequently described how their church brought in a new pastor who was less familiar with the FAN program (“We had a complete change in leadership, from head pastor down. FAN disappeared as a result.”) Some also mentioned that their original FAN coordinator left the position, which made it difficult for the FAN committee to continue to plan events and programs. Other leaders simply described a lack of internal support, defined as issues with being unable to plan or be consistent with church activities for the program, as a barrier to PA and HE maintenance.

### Facilitators

Several themes consistently emerged to describe facilitators for maintaining the PA and HE components of FAN (Table 3). Healthy opportunities, communication, and consistency were the most frequently identified facilitators for both PA and HE maintenance. External support and leadership were also cited as PA and HE facilitators in both phases. In general, church leaders described more facilitators for PA than for HE: Other less commonly cited PA facilitators in both phases were fun and tailoring. In Phase 1 only, internal support was mentioned as both a PA and HE facilitator.

**Table 3:** Themes and select responses to physical activity and healthy eating maintenance facilitators

Theme	Physical activity facilitators (select quotations)	Ph 1 Count	Ph 2 Count	Healthy eating facilitators (select quotations)	Ph 1 Count	Ph 2 Count
Healthy opportunities	We walked around the church. We had a fast group, then a middle group, and then the slow group. And we'd walk so many times around the church. And then some of them walked a good ways from the church too	25	45	Including healthy choices with the regular offerings and encouraging more healthy eating with providing those choices	28	44
	Consistency in planning physical activity as a part of our weekly children's and youth gatherings			Fruits and vegetables are promoted at all church events		
	Encouraging persons to walk weekly and adjusting the location to out of doors when weather permitted. Tying our activity to other missions such as Walking for a local Mission and walking in the Alzheimer's walk			Every meal we did we baked instead of fried. We did not use pork or fat back to cook vegetables. We always serve fruit trays and included water		
Communication	Daily texts to remind of daily walk, and daily emails	18	22	Posting information about healthy eating on the bulletin board. Reminding congregation that fruits and vegetables are served to be eaten	25	41
	Bringing attention to physical activity through emails and handouts			Consistent messages in our church's monthly newsletter and bulletin inserts. The food co-op encourages including fruits and vegetables in diet		
Consistency	We have a FAN moment each Sunday during the Worship Service. We have a faith walk most Sundays following service where we walk around the church. We have a walking club called the Christian Steppers who Walk once a week- 1.6 miles	16	25	Weekly notices to eat healthy	16	28
	Our monthly meetings, announcements in church, and every so often it comes across the pulpit after the sermon			Consistent about what we've provided at Wednesday meals, encouraged youth		
				Focusing on healthy eating at monthly fellowship luncheon and healthy eating programs		
External Support	The material that that we received in the mail was very helpful, in making us aware that we needed to get up and move	6	15	Bulletin board with FAN info, starting new program for youth, and FAN handouts	9	20
				Using FAN recipes for events involving food		
Leadership	The coordinator would do exercise between Sunday school and morning service	10	15	The coordinator would provide healthy snacks and salad lunches	8	13
	The Pastor's communication of it and personal example			The Pastor communicating it from the pulpit		
Fun & Tailoring	People just got excited about pickle ball, tied in to FAN, got others excited	9	29	*Not a prominent theme for healthy eating*		
	They loved the FAN training, how you get up and pray and step in place, they kept the in motion, most members are older. But still finding ways that support the elderly and young					
	Consistency in planning physical activity as a part of our weekly children's and youth gatherings					
Internal support (Phase 1 only)	When we work as a group, it helps. realizing other churches need to commit with them for physical activity	13	10	The Committee kept it before the church and they kept in touch with the FAN coordinator	8	11
	Try to get together every three months to do walking, and different kind of exercises			Motivation. If we do an activity we encourage them to participate		

Themes were considered prominent if they featured in more than 10% of responses for each question. Ph 1 = Phase 1. Ph 2 = Phase 2

#### Healthy Opportunities

The most common PA and HE facilitator theme among church leader responses was healthy opportunities. Healthy opportunities were defined as church leaders’ ability to provide healthful options or activities to church members during regular church activities. When describing PA opportunities, church leaders spoke about exercise breaks that were integrated into church programming. They also discussed regular PA-focused events that the church hosted, such as a walking group or fitness classes. HE opportunities cited by church leaders included changes to food preparation methods or the provision of healthy options, namely fruits and vegetables, for church-based meals or snacks (“Fruits and vegetables are promoted at all church events.”).

#### Communication

Communication referred to the act of communicating FAN program materials or information. Church leaders described communication as a facilitator for both PA and HE maintenance, mainly through education and regular messaging at services and meetings. For education, some church leaders mentioned sharing PA and HE tips in weekly church bulletins, in emails, and at church meetings. Other leaders described how their church communicated health tips through different mediums, such as text messages, or word of mouth (“Daily texts to remind of daily walk, and daily emails.”) Messaging often referred to either the pastor or FAN coordinator making announcements to promote PA and HE or to share healthy tips during church services.

#### Consistency

Consistency emerged as a prominent facilitating theme for both PA and HE maintenance and referred to regularly held church activities or initiatives that supported PA and HE. When describing consistency, pastors and FAN coordinators discussed both regular communication and frequently scheduled activities as facilitators for PA and HE. Consistent communication was mostly described as weekly updates or information sharing through announcements at services or in bulletins. Regularly scheduled activities were consistent healthy options provided by the church, such as healthy options at all church meals, monthly or annual health challenges, or a weekly exercise class (“We have a faith walk most Sundays following service where we walk around the church. We have a walking club called the Christian Steppers who walk once a week—1.6 miles.”).

#### External Support

Church leaders referred to external support, or material/intangible support provided by outside entities, mostly as a HE facilitator. In most cases, leaders referenced the FAN handouts and monthly materials that were created by the research team and provided as a part of the program. Leaders specifically mentioned their use of provided bulletin inserts to promote HE, as well as healthy recipe ideas (“Using FAN recipes for events involving food.”).

#### Leadership

Leaders’ actions were discussed by many pastors and FAN coordinators mostly as PA facilitators. Many leaders mentioned how the pastor’s modeling of PA (i.e., leading the church in a walking group) was encouraging to members. Other leaders described how the effective leadership and enthusiasm of the FAN committee contributed to participation in PA events among members (“The coordinator would do exercise between Sunday school and morning service.”).

#### Fun and Tailoring (PA Only)

The themes fun and tailoring emerged in both phases as facilitators to PA maintenance. For fun, leaders described how the FAN committee engaged members in activities such as contests, games, or sports leagues as PA opportunities (“People just got excited about pickle ball, tied in to FAN, got others excited.”) Some leaders also described how the FAN committee offered novel physical activities, such as historical walking tours or dance classes, to engage more members. When discussing tailoring, pastors and FAN coordinators spoke mostly about different PA opportunities that were adapted by age group. Some leaders spoke about chair exercise classes (or otherwise modified exercise classes) for older members; while, others described PA opportunities tailored specifically to youth.

#### Internal Support (Phase 1 Only)

Internal support, or structures/individuals within the church that promoted FAN activities, was mentioned by many church leaders in Phase 1 as both a PA and a HE facilitator. Internal PA support was often described as encouragement to be active, either from the FAN committee or among church members (“When we work as a group, it helps, realizing other churches need to commit with them for physical activity.”) For HE, many church leaders mentioned ways in which they held their members accountable to the HE parts of FAN, such as continually presenting FAN HE information to church members or encouraging members to try healthy options at church meals (“The committee kept it before the church and they kept in touch with the FAN coordinator.”).

### Support for and Intent to Maintain FAN

In Phase 1, the mean for the extent to which PA components were supported by the church between months 12–24 was 2.14 (pastors) and 2.91 (FAN coordinators), which corresponded to “there has been little or no support” (score of 2) and “there has been some support” (score of 3) (Table 4). Phase 1 church leaders reported scores of 2.28 (pastors) and 3.22 (FAN coordinators) for the extent to which FAN HE components were supported by the church (a score of 4 equated to “there has been strong support”). Mean responses for the likelihood that the church will continue PA activities in upcoming year were 3.39 (pastors) and 3.31 (FAN coordinators); while, mean responses for the likelihood that the church will continue HE activities in upcoming year were 3.50 (pastors) and 3.33 (FAN coordinators). These responses all fell between “somewhat likely” (score of 3) and “very likely” (score of 4).

**Table 4:** Support for FAN during 12–24 months and intent to maintain FAN 24–36 months

	Phase 1 (County-wide initiative)		Phase 2 (State-wide initiative)	
	Pastors (*n* = 28) Mean (*SD*) Range	FAN Coordinators (*n* = 33) Mean (*SD*) Range	Pastors (*n* = 53) Mean (*SD*) Range	FAN Coordinators (*n* = 67) Mean (*SD*) Range
Extent to which physical activity components were supported by church over the past year	2.14 (0.52)	2.91 (0.64)	2.00 (0.78)	2.82 (0.82)
	(1.0–3.0)	(1.0–4.0)	(1.0–3.0)	(1.0–4.0)
Extent to which healthy eating components were supported by church over the past year	2.28 (0.46)	3.22 (0.71)	2.10 (0.75)	3.04 (0.81)
	(2.0–3.0)	(1.0–4.0)	(1.0–3.0)	(1.0–4.0)
Likelihood that church will continue physical activity activities in upcoming year	3.39 (0.78)	3.31 (0.90)	3.09 (0.99)	2.93 (1.06)
	(1.0–4.0)	(1.0–4.0)	(1.0–4.0)	(1.0–4.0)
Likelihood that church will continue healthy eating activities in upcoming year	3.50 (0.74)	3.33 (0.89)	3.10 (0.98)	2.86 (1.07)
	(1.0–4.0)	(1.0–4.0)	(1.0–4.0)	(1.0–4.0)

For support, response options were “The church has been resistant” (1), “There has been little or no support” (2), “There has been some support” (3), “There has been strong support “(4). For likelihood to continue, response options were “very unlikely” (1), “Somewhat unlikely” (2), “Somewhat likely” (3), “Very likely” (4)

In churches where pastors had received FAN training, pastor perception of church likelihood to continue FAN HE activities was more favorable than among those who had not received training (*r* = 0.56, *ES* = large). Also, in churches that reported having a health ministry, pastor perception of church support for FAN PA activities was more positive than in those without a health ministry (*r* = 0.44, *ES* = medium). Church size and predominant race of congregation were not associated with pastors’ or FAN coordinators’ perceptions of support for or intent to maintain FAN (all *p* values > 0.05) in Phase 1.

In Phase 2, the mean for the extent to which PA components were supported by the church between months 12–24 was 2.00 (pastors) and 2.82 (FAN coordinators), which fell between the response options “there has been little or no support” and “there has been some support” (Table 4). For HE support, Phase 2 church leaders reported scores of 2.10 (pastors) and 3.04 (FAN coordinators). Mean responses for the likelihood to continue PA activities in upcoming year were 3.09 (pastors) and 2.93 (FAN coordinators), while mean responses for the likelihood that the church will continue HE activities in upcoming year were 3.10 (pastors) and 2.86 (FAN coordinators). These responses corresponded with a rating of “somewhat likely” (score of 3).

Predominant race of the congregation was the only church-level variable consistently associated with perceptions of support for or intent to maintain FAN in Phase 2. Pastor perception of church support of HE activities (*r* = 0.33, *ES* = medium), church support of PA activities (*r* = 0.27, *ES* = small), likelihood to continue HE activities (*r* = 0.34, *ES* = medium), and likelihood to continue PA activities (*r* = 0.37, *ES* = medium) were more favorable in predominantly Black/African American churches relative to white churches. A similar pattern emerged for the FAN coordinator perceptions, although *p* values did not reach *p* < 0.05. Finally, in churches that reported having a health ministry, pastor perception of church support of PA was more positive (*r* = 0.31, *ES* = medium) than in churches without a health ministry. Member size and pastor training were unrelated to all variables.

## Discussion

Despite the promise of faith-based settings for health promotion, very little is known about factors that may support or impede long-term program maintenance in churches. Few studies to date have examined long-term program maintenance in a faith-based setting (Allicock, 2013; Allicock et al., 2012; Scheirer et al., 2017; Wilcox et al., 2020); even fewer have qualitatively examined barriers and facilitators to program maintenance in faith-based settings. Our study described church leaders’ perceived barriers to and facilitators of maintaining the PA and HE components of FAN and examined church leaders’ perceived support for FAN during the follow-up period and intent to maintain FAN going forward. Understanding these factors may inform the tailoring of future faith-based health promotion programs in similar settings and may also be useful in guiding technical program assistance provided to church leaders in similar settings.

There were differences in reported barriers for PA and HE. In both phases, leaders frequently reported that resistance to change impeded HE maintenance, but not PA. When describing resistance to change as a barrier to HE, leaders discussed how individual and cultural preferences contributed to waning support for healthier dietary choices. The FAN program encouraged churches to adapt favorite recipes and to make dietary changes gradually. Nonetheless, these results suggest that more emphasis needs to be placed on these messages, and church leaders need more tools for how to overcome resistance. Because the importance of leader role modeling has been identified as influential in member HE behaviors specifically (Baruth et al., 2011; Bopp & Fallon, 2011), a possible intervention approach may be to focus on church leader HE behaviors. As previous studies have found that church leaders may be at an increased risk of obesity and obesity-related chronic disease (Baruth et al., 2014; Proeschold‐Bell & LeGrand, 2010), an intervention approach that aims to improve church leader health behaviors may yield benefits for both church leaders and their congregants. Previous studies have also used lay health advisors to model health behavior (Kennedy et al., 2005; Wilcox et al., 2007), which may also be a viable option for HE behavior modeling in faith-based settings.

For PA, leaders described church characteristics as a barrier to maintenance. Age of members was discussed in many responses as leaders observed that older members were less likely to feel willing to or capable of participating in PA programs. This finding is not surprising given that existing research has identified a decline in PA with age and the importance of self-efficacy as a determinant of older adult PA (French et al., 2014; Keadle et al., 2016). However, these results may also reflect church leader biases about older adults or the need for churches to get more instruction in adapting PA to an older population.

Overall, church leaders in Phases 1 and 2 reported similar facilitators to PA and HE maintenance at 24 months: healthy opportunities, communication, and consistency. Other studies have corroborated church leaders’ emphasis on healthy opportunities and activities in their churches, and the importance of healthy opportunities at church to health behavior change among members. A 2011 study of over 800 faith leaders found that the majority were offering health and wellness activities (Bopp & Fallon, 2011). Another study of 24 Black churches in North Carolina found that providing fruits and vegetables at church meals was associated with greater fruit and vegetable intake among church members (Campbell et al., 2000). Communication has also been previously identified as important to health behavior change in faith-based interventions: One study found that sharing HE messages, either through announcements by church leaders or in print materials, was associated with better dietary practices among church members (Baruth et al., 2011).

In a previous study examining church leaders’ reported barriers and facilitators before and after the implementation of the FAN program, Bernhart et al. (2019) found that the most cited barriers to implementation were no anticipated barriers, resistance to change, church characteristics, and lack of participation/motivation. Although nothing/no barriers appeared as a theme in our study, particularly for HE, church leaders mentioned more barriers overall to 24-month maintenance of the FAN program as compared to 12-month implementation. This finding may reflect the difficulty of long-term program maintenance for churches. However, resistance to change, church characteristics, and lack of participation/motivation were all important barriers to 24-month maintenance, which suggests that there may be opportunities to tailor interventions around these factors to enhance both program implementation and long-term maintenance. In contrast, Bernhart et al. found that the most common facilitators to the FAN program implementation were internal support, leadership, and communication while our study identified healthy opportunities, communication, and consistency. It may not be surprising, however, that factors pertaining to church support and leadership are important to initial program implementation while factors like the consistent provision of healthy opportunities are more important to long-term program sustainability. Importantly, communication appears to be a key facilitator in both program implementation and maintenance and may be an area of opportunity for future interventions in faith-based settings.

Of the available qualitative studies that have examined long-term health program maintenance in other settings, similar barriers and facilitators to those in the current study have emerged. In a 2003 study examining health promotion program maintenance in schools, staff and leadership support (and turnover in leadership positions) were particularly important to program success (Lytle et al., 2003). More recently, in an analysis of community PA programs for older adults, the presence of program champions was an important aspect of maintenance and long-term sustainability, as were partnerships to support the program and a financial plan to sustain activities (Estabrooks et al., 2011). These results align with several of the prominent barriers and facilitators found in our study and point to the common challenges to organizational maintenance across settings. They also underline the need for more analysis of long-term health promotion program maintenance in general, as it may be possible to share insights and improve programs across settings.

At 24 months in both phases, pastors indicated that there had been “little or no support” or “some support” within the church for both the PA and HE components of FAN; while, FAN coordinators indicated that there had been “some support” or “strong support.” The differences in pastor and FAN coordinator perspectives may be explained by the degree to which they were involved in program implementation. FAN coordinators would have had more hands-on experience implementing the PA and HE components of FAN; while, pastors may have based their responses to these questions on their perceptions of FAN overall. This also aligns with our finding that pastor perception of church support for FAN PA activities was more positive in churches that reported having a health ministry (Phase 1 only).

For likelihood of maintaining PA and HE FAN activities in the upcoming year, pastors in both phases and FAN coordinators in Phase 1 reported that it was “somewhat likely” or “very likely” that the church would continue activities, while FAN coordinators in Phase 2 reported that it was “somewhat likely” that the church would maintain PA and HE activities. Again, this may be attributable to differences in leader perspectives in Phase 2, particularly as a lack of leadership was commonly cited as a barrier to FAN maintenance in Phase 2. Nonetheless, these results align with Wilcox et al.’s findings that church leaders in Phases 1 and 2 reported significantly greater implementation of both PA and HE FAN components at 12 and 24 months compared to baseline and that most churches maintained at least one FAN component at 24 months (2020). Further, leaders from Phase 1 churches who took part in the 24-month surveys represented predominantly Black/African American congregations, whereas those from Phase 2 churches were evenly split between predominantly Black/African American and white congregations. Predominant congregant race was also consistently associated with pastor perceptions of support for FAN activities and likelihood to maintain: all scores among Phase 2 pastors were more favorable in majority African American churches compared to majority white churches. This aligns with the overarching role of promoting health in Black churches that is more well-established, and consistent with a broad role that the church plays in the lives of Black/African American individuals (Brewer & Williams, 2019).

### Limitations

This study had several limitations. First, data were collected through brief telephone surveys instead of through in-depth interviews, which would have provided the opportunity for additional probes to participant responses and may have yielded richer data. Second, our sample was limited to churches in SC, and the results of this study may not be generalizable to churches in other geographic areas. Third, the survey completion rates at 24 months were less than ideal for Phase 2 churches (87% of churches; 75% of FAN coordinators, 58% of pastors). Despite these limitations, this study was strengthened by several factors. First, the study included perspectives from two different samples of church leaders in two different phases of FAN, which allowed for comparison of emergent themes between groups. Additionally, to our knowledge it is the only qualitative study to date to examine barriers and facilitators to long-term program maintenance in a faith-based setting. As more research on factors influencing program maintenance is needed to inform program design and sustainability, this study adds an important dimension to the existing health promotion program maintenance literature.

## Conclusions

Future research should build upon the lessons learned from this study to tailor faith-based health promotion programs. For example, programs may build upon FAN by focusing on a given facilitating component, such as church-based communication of health information. In addition, researchers may want to consider overcoming maintenance barriers such as resistance to change by engaging church leadership in health behavior modeling. By emphasizing facilitators and working to reduce barriers to PA and HE maintenance, faith-based health promotion programs have the potential to improve long-term program sustainability in churches and improve health outcomes among members at-risk of chronic disease.
